# An mRNA-Based Respiratory Syncytial Virus Vaccine Elicits Strong Neutralizing Antibody Responses and Protects Rodents Without Vaccine-Associated Enhanced Respiratory Disease

**DOI:** 10.3390/vaccines13010052

**Published:** 2025-01-09

**Authors:** Jianglong Li, Haiyan Long, Shaoyi Chen, Zhendong Zhang, Shuang Li, Qi Liu, Jun Liu, Jiaru Cai, Liping Luo, Yucai Peng

**Affiliations:** Liverna Therapeutics Inc., Zhuhai 519000, China; lijianglong@live-rna.com (J.L.); longhaiyan@live-rna.com (H.L.); chenshaoyi@live-rna.com (S.C.); zhangzhendong@live-rna.com (Z.Z.); lishuang@live-rna.com (S.L.); liuqi@live-rna.com (Q.L.); liujun@live-rna.com (J.L.); caijiaru@live-rna.com (J.C.); luoliping@live-rna.com (L.L.)

**Keywords:** RSV, mRNA vaccine, F protein, LVRNA007, viral challenge study

## Abstract

Background: Respiratory syncytial virus (RSV) causes the most common type of severe lower respiratory tract infection worldwide, and the fusion (F) protein is a target for neutralizing antibodies and vaccine development. This study aimed to investigate the immunogenicity and efficacy of an mRNA-based RSV vaccine with an F protein sequence. Methods: We designed an mRNA construct encoding a modified RSV F protein, which was further developed into an LNP-encapsulated mRNA vaccine (LVRNA007). LVRNA007 was administered to mice and cotton rats, followed by immunogenicity analysis and viral challenge studies. Protection of rodents from the viral infection was evaluated based on the presence of the virus in the lung and pathological examination of respiratory tissues. Results: LVRNA007 induced robust humoral and cellular immune responses in both mice and cotton rats, with neutralization antibody levels in the immunized animals maintained at high levels for over one year. Vaccination of LVRNA007 also protected the rodents from RSV challenge, judged by the much decreased virus titer and the pathological score in the lung tissue. In addition, no vaccine-enhanced disease (VED) phenomenon was observed with LVRNA007 vaccination. Conclusions: Based on the preclinical immunogenicity and efficacy data, LVRNA007 could be a potential promising vaccine for prophylaxis of RSV infection.

## 1. Introduction

Respiratory syncytial virus (RSV) is one of the main agents of lower respiratory tract infection in infants and the elderly [1,2]. RSV infections cause more than 3 million hospitalizations and almost 120,000 deaths in children 5 years of age and under annually. Of these, approximately 44% of hospitalizations and 46% of in-hospital deaths are among infants up to 6 months of age [3]. RSV is a negative-sense, single-stranded RNA virus with two subtypes, RSV A and RSV B, that belongs to Pneumoviridae (genus Orthopneumovirus) [4,5,6,7]. The RSV genome is 15.2 kb in length and encodes a total of 11 proteins: nonstructural protein 1 (NS1), NS2, nucleoprotein (N), phosphoprotein (P), matrix (M) protein, small hydrophobic (SH) protein, attachment (G) glycoprotein, fusion (F) glycoprotein, M2-1 protein, M2-2 protein, and large polymerase subunit (L) [8,9,10]. The F protein is a metastable class I fusion protein that mediates viral fusion with host cell membranes [8,9]. Furthermore, the F protein is highly conserved across RSV types A and B, elicits both neutralizing antibodies and cytotoxic T-lymphocyte responses, and represents the main target for vaccine development and monoclonal antibodies [11,12,13].

An effective intervention that prevents RSV disease in all infants is a major public health priority [14]. RSV vaccine development has become more important. Traditional vaccines are typically based on live viruses, inactivated/attenuated viruses, or subunit proteins derived from pathogens, and they have induced effective immune responses [15]. However, these vaccines present a number of associated safety concerns. Live attenuated RSV vaccines that are insufficiently attenuated, or that are highly attenuated but insufficiently immunogenic [16,17,18], have difficulty achieving a balance between attenuation and immunogenicity in naïve children and infants [9,15,19]. A formalin-inactivated whole-virus RSV vaccine was reported to cause vaccine-enhanced disease in infants during a series of clinical trials [20,21,22,23]. Subunit vaccines have been avoided in RSV-naïve children due to concerns of enhanced respiratory disease associated with protein-based vaccines [15,24]. Each type of vaccine and vaccine platform has different challenges and opportunities, and a safe and effective RSV vaccine remains necessary.

Messenger RNA (mRNA) vaccine technology offers the promise of providing rapid antigen design and scalable production [25,26]. mRNA vaccines begin with gene sequences, which eliminate the need to produce and purify proteins and use cell culture systems [27]. mRNA vaccines can provide a safe and controllable pattern of gene expression without the risk of nucleic acid integration into the host genome [28]. The in vitro transcribed (IVT) mRNA would be susceptible to nuclease breakdown when injected into animals and lead to innate immunogenicity comparable to that experienced when infected by a pathogen [29,30]. Pseudouridine can be used to replace uridine in the IVT mRNA, which enhances RNA stability, decreases the anti-RNA immune response, and was used for mRNA COVID-19 vaccines [31,32]. However, to avoid immune suppression, mRNA vaccines with a lower percentage of N1-methyl-pseudouridine modification should be used for future clinical trials for cancers [33]. N1-methylpseudouridylation of mRNA has been reported to lead to +1 ribosomal frameshifting [34], suggesting that the sequence of mRNA vaccines should be checked to avoid mistranslation. Although mRNA vaccines have been shown to have some detrimental effects, they have attracted significant interest and offered a lot of hope as one of the cutting-edge, new technologies. In response to the severe acute respiratory syndrome coronavirus 2 (SARS-CoV-2) pandemic, mRNA vaccines were rapidly developed and manufactured [35]. Recently, mRNA vaccines have been reported to be applied to various infectious diseases, including RSV [36].

The current RSV vaccine candidates that mainly focus on targeting the pre-F conformation have elicited superior neutralizing antibody responses [37]. mRNA vaccines for RSV have been widely developed, as an engineered coding sequence can produce stabilized F protein conformations [38]. For example, an mRNA-based RSV pre-fusion F protein vaccine (mRNA-1777) was shown to promote neutralizing antibodies and trigger a strong humoral response [39]. Further sequence optimization led to vaccine mRNA-1345, with a more stable pre-fusion form of the RSV F protein [40]. The lipid nanoparticle (LNP) is a versatile platform for the efficient delivery of mRNA molecules. LNPs are safe and effective for repeated intramuscular delivery, as validated in clinical trials [41,42,43,44]. Here, we developed an LNP-encapsulated chemically modified mRNA vaccine (LVRNA007) encoding a pre-fusion form of the RSV F protein and evaluated its immunogenicity and efficacy in rodent models.

## 2. Materials and Methods

### 2.1. Vaccines

The mRNA vaccine (LVRNA007) was designed based on the RSV F sequence of the RSV A2 strain (GenBank: LY751597.1), which stabilized the pre-fusion conformation with modifications to optimize DS-Cav1, described by McLellan et al. [45]. The vaccines were produced based on the Liverna Therapeutics platform (China patent ZL201911042634.2). Briefly, plasmids containing the open reading frame flanked by the 5′- and 3′-UTRs and poly-A sequences were linearized, and the plasmid production process was well-controlled to prevent nucleic acid contamination. The mRNAs were synthesized through in vitro transcription using T7 RNA polymerase (Novoprotein Scientific Inc., Suzhou, China) with the replacement of uridine by N1-methyl-pseudouridine. DNase I (Novoprotein Scientific Inc., Suzhou, China) was added to terminate the in vitro transcription reaction. After the transcription reaction, the mRNAs were purified using an oligo-dT affinity column (Sepax Technologies, Inc., Suzhou, China) and tangential flow filtration (Repligen Corporation, Waltham, MA, USA). The purified mRNAs were encapsulated in LNPs containing 49% DLin-MC3-DMA, 7% 1,2-distearoyl-sn-glycero-3-phosphocholine, 41% cholesterol, and 3% polyethylene glycol lipid (mol%). Finally, the products were stored prior to injection into animals.

The transmission electron microscopy (TEM) method used to analyze the morphology of the nanoparticles was previously described [46]. Briefly, approximately 5 µL of nanoseeds was dropped onto a carbon-coated 400-mesh copper grid. Under atmospheric conditions, the sample was rendered hydrophilic by glow discharge (EmiTech, Paris, France) for 1 min at 25 mV. Then, the sample was stained with 2% uranyl acetate. After air-drying, the nanoparticles were observed and photographed at an acceleration voltage of 80 kV by a TEM (Hitachi, HT7800, Tokyo, Japan).

### 2.2. Verification of F Protein Expression

HEK293T cells were grown in Dulbecco’s modified Eagle medium (DMEM) containing 10% fetal bovine serum (FBS). Cells were transfected with mRNA using Lipofectamine^®^ MessengerMAX™ transfection reagent (Invitrogen, Carlsbad, CA, USA) for 48 h. Cells were collected and lysed, and the extracted protein was separated through sodium dodecyl sulfate–polyacrylamide gel electrophoresis. The protein was then transferred onto polyvinylidene difluoride membranes. The membranes were blocked with 5% skimmed milk for 1 h at room temperature and incubated with primary antibodies against RSV-F (1:1000, Sino Biological Inc., Beijing, China) and GAPDH (1:10,000, Abcam, Cambridge, MA, USA) at 4 °C overnight. Afterwards, the membranes were incubated with a secondary antibody, HRP-conjugated goat anti-rabbit IgG (1:5000, BBI Life Sciences, Cambridge, UK), for 1 h at room temperature. The bands were visualized with an enhanced chemiluminescence substrate (Perkin-Elmer, Waltham, MA, USA) using a ChemiDoc XRS+ system (Bio-Rad, Hercules, CA, USA).

### 2.3. Flow Cytometry Analysis

After transfection, the HEK293 cells were collected and labeled with F protein antibodies, including anti-antigenic site Φ (Vazyme, Nanjing, China), anti-trimeric site Φ (Vazyme, Nanjing, China), and anti-site IV (Vazyme, Nanjing, China), for 15 min in the dark. The cells were analyzed using a CytoFLEX flow cytometer (Beckman Coulter, Indianapolis, IN, USA), and the flow cytometry data were processed using FlowJo software v10 (Tree Star, Inc., Ashland, OR, USA).

### 2.4. Vaccination and Viral Challenge in Mice

BALB/c mice (6–8 weeks) were purchased from Shanghai Jihui Laboratory Animal Care Co., Ltd. (Shanghai, China). The IACUC approval number was SOP-QDACU-FAC-008-202301.

To study the durability of the immune response, mice were randomly distributed into three groups (*n* = 10/group) according to their treatment: (i) LVRNA007 (5 μg), (ii) LVRNA007 (15 μg), and (iii) empty LNP (as the negative control). Mice were administered LVRNA007 (5 μg or 15 μg) or LNP intramuscularly (i.m.) on days 0 and 28. Serum samples were collected every 28 days for serological analysis. Antibody tests were continued over 364 days. Cytokines were examined in lymphocytes 90 days after the second vaccination.

In the challenge study, mice were randomly distributed into five groups (*n* = 6/group) according to their treatment: (i) LNP (as the negative control), (ii) FI-RSV (formalin-inactivated RSV vaccine), (iii) LVRNA007 (5 μg), (iv) LVRNA007 (15 μg), and (v) Health (without viral infection). Mice were immunized i.m. with LVRNA007 (5 μg or 15 μg, days 0 and 21), FI-RSV (0.05 μg, days 0 and 21), or LNP (50 μL, days 0 and 21). Two weeks after the second immunization, mice were challenged intranasally with 5 × 10^5^ PFU (plaque-forming unit) of RSV A2 (0.05 mL). Mice in the Health group did not receive any treatment. Body weight was recorded and calculated in all groups 0, 7, 14, 21, 28, 35, 36, 37, 38, 39, and 40 days post-immunization. Blood samples were collected on day 35 and subsequently stored at −20 °C until analysis. Five days following the RSV challenge, mice were euthanized, and lung tissue samples were collected for viral titration and histopathology.

### 2.5. Vaccination and Viral Challenge in Cotton Rats

Cotton rats (3–7 weeks) were obtained from SPF Biotechnology Co., Ltd. (Beijing, China). The IACUC approval number was SOP-QDACU-FAC-031-202301. Cotton rats were randomly distributed into five groups (*n* = 8/group) according to their treatment: (i) LNP, (ii) RSV live virus, (iii) FI-RSV, (iv) LVRNA007 (5 μg), and (v) LVRNA007 (25 μg). Cotton rats were immunized i.m. with LVRNA007 (5 µg or 25 µg), FI-RSV (0.05 μg), or LNP (mock, 125 µL) on days 0 and 28. For the RSV live virus group, cotton rats received intranasally 5 × 10^5^ PFU of RSV A2 (0.1 mL) on day 28. All cotton rats were challenged intranasally with 1 × 10^6^ PFU of RSV A2 delivered in a 0.1 mL volume on day 56. Body weight was monitored and calculated on days 0, 7, 14, 21, 28, 35, 42, 49, 56, 57, 58, 59, 60, and 61. Blood samples were drawn on days 28 and 56 for serological assays. On day 5 post-challenge, cotton rats were euthanized. Turbinate bones were harvested for virus quantification, and lungs were isolated for viral titration, histopathology, and cytokine mRNA analysis. Lung washes were harvested by flushing the lungs. The lavage fluid was centrifuged to remove cells and stored at −20 °C.

### 2.6. Analysis of Cellular Immune Responses

Spleen lymphocytes from immunized animals were collected and resuspended in RPMI 1640 medium containing 10% FBS. Then, cells (4 × 10^6^) were seeded on a 24-well flat-bottom tissue culture plate, and a synthesized peptide library of RSV-F (524 amino acid residues, 103 overlapping 15-mer peptides with 10-amino-acid overlap) was added. After incubating for 72 h at 37 °C, the cell supernatants were collected, and interferon (IFN)-γ and interleukin (IL)-2, IL-4, and IL-10 levels were determined using enzyme-linked immunosorbent assay (ELISA) kits (Neo Bioscience, Guangzhou, China) according to the manufacturer’s instructions.

### 2.7. Serum IgG Measurement

Serum samples were collected from mice or cotton rats, and serum F-specific IgG was detected using indirect ELISA. Briefly, 96-well ELISA plates were coated with purified recombinant RSV F protein (0.5 μg/well) at 4 °C overnight and blocked with 3% skim milk for 1 h at room temperature. The RSV F-coated plates were incubated with serially diluted serum samples from mice or cotton rats for 1 h at room temperature. After washing three times with PBST, plates were covered with HRP-conjugated goat anti-mouse or rat IgG secondary antibody (1:3000, Invitrogen, Waltham, MA, USA) for 1 h. Serum F-specific antibody titers were measured at 450 nm. The antibody endpoint titer was based on the highest dilution that gave an absorbance reading twice that of the naïve group without dilution.

### 2.8. Serum Neutralization Assay

Serum samples were inactivated by heating at 56 °C for 30 min and then serially diluted with DMEM. The diluted serum was added to 96-well plates and mixed with 50 PFU of an RSV virus suspension at 37 °C for 1 h. Then, Hep-2 cells were inoculated with 100 µL of the suspension in duplicate. After incubation for 48 h at 37 °C in a 5% CO_2_ incubator, the medium was removed, and 0.9% methylcellulose medium was added. Three days later, cells were fixed and stained with 0.1% crystal violet. RSV-specific neutralizing antibody levels were determined and expressed as the reciprocal of the serum dilution that produced a 50% reduction in PFU relative to the number of plaques without the addition of antibody.

### 2.9. Cotton Rat Cytokine Expression Analysis

The cytokine expression in cotton rats was determined using real-time PCR. Briefly, total RNA was isolated using Trizol reagent and converted to complementary DNA (cDNA) using a reverse transcription system (Promega, Beijing, China). Real-time PCRs were performed to determine the cytokine expression using a QuantiFast SYBR Green PCR kit (Qiagen, Shanghai, China). The final primer concentration for amplifying the cytokine mRNA was 0.5 μM. The real-time PCR conditions were as follows: 1 cycle at 95 °C for 3 min, followed by 40 cycles at 95 °C for 10 s, 60 °C for 10 s, and 72 °C for 15 s. The relative expression levels of different cytokines were calculated using the 2^−ΔΔCT^ method. β-Actin was used as the reference gene.

### 2.10. RSV Plaque Assay

The lung tissue was collected and subjected to homogenization with a BeadBeater (Biospec Products, Bartlesville, OK, USA), as described previously [47]. Hep-2 cells (2 × 10^5^ cells/well) were seeded on 24-well plates and incubated with serial dilutions of RSV prepared in lung supernatants. After adsorption for 1 h at 37 °C, the liquid was aspirated, and the cells were overlaid with 0.8% methylcellulose. Five days later, cells were fixed with glutaric dialdehyde and stained with crystal violet. Viral plaques were visualized and counted. Virus titers were expressed as PFU/g of tissue.

### 2.11. Histopathology

Lungs were collected, fixed in 10% neutral buffered formalin, and embedded in paraffin. The tissue was sectioned and stained with hematoxylin and eosin (HE). Four parameters associated with lung pathological changes were evaluated: perivasculitis, peribronchiolitis, alveolitis, and interstitial pneumonia. Each of these parameters was scored separately using a 0–4 severity scale (0 = absent and 4 = maximum/severe). All slides were evaluated by an independent pathologist in a random and blinded manner.

### 2.12. Statistical Analysis

The data were presented as the mean ± standard deviation (SD) and statistically analyzed using GraphPad Prism (8.0) software (San Diego, CA, USA). Comparisons among groups were conducted using ordinary one-way or two-way analysis of variance (ANOVA), followed by Tukey’s test. A *p* value less than 0.05 was considered statistically significant.

## 3. Results

### 3.1. Design and Expression of the mRNA Antigen

**Figure 1:**
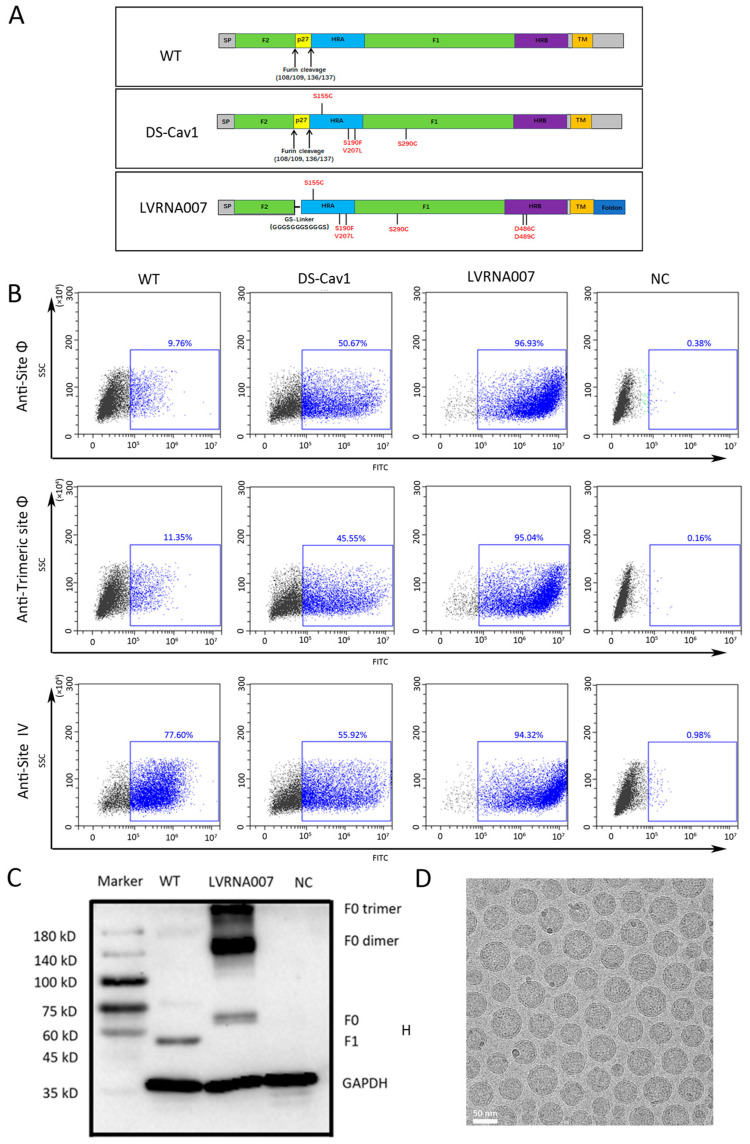
Design and expression of the mRNA antigens. (**A**) Schematic diagram of mRNA vaccine constructs (wild, DS-Cav1, or LVRNA007). SP, signal peptide. p27, p27 peptide. HRA, heptad repeat A. HRB, heptad repeat B. TM, transmembrane peptide. (**B**) HEK293T cells transfected with in vitro transcription (IVT) mRNA bound to antibodies against the antigenic site Φ, the trimeric site Φ, and site IV were analyzed using flow cytometry. (**C**) HEK293T cells were transfected with mRNA, and Western blotting was conducted to detect F0, F1, and GAPDH proteins. (**D**) Spherical nanoparticles were observed using a transmission electron microscope. Scale bar, 50 nm.

The F protein induces neutralizing antibodies that are conserved and elicit better cross-protection against different RSV strains [24,48,49]. Research on the humoral immune response to natural RSV infection has revealed that the majority of human antibodies target the pre-fusion conformation of the F protein [50,51]. A S155C-S290C double mutant (DS) and a S190F-V207L pair (Cav1) variant have been reported to form stable RSV F trimers and retain antigenic site Φ [45]. The disulfide bond introduced with the D486C/D489C mutations contributed to retaining the structure of the lower part of the molecule and improved the stability of the pre-fusion trimer [52]. In the current study, an mRNA construct encoding the RSV F protein, which stabilized the pre-fusion conformation, was designed to develop into an mRNA vaccine (LVRNA007). The LVRNA007 construct incorporated S155C-S290C, S190F-V207L, and D486C/D489C mutations to form stable RSV F trimers. In addition, the p27 peptide had a negative effect on trimerization even when a trimerization motif was present [53]. We removed amino acids 98–144 of the DS-Cav1 construct (including the Furin cleavage sites, the p27 peptide, and part of the fusion peptide), which we replaced with a flexible linker. Furthermore, the T4 phage fibritin trimerization domain (foldon) was appended to the C terminus of the RSV F ectodomain to facilitate F trimerization (Figure 1A). As shown in Figure 1B, flow cytometry analysis demonstrated that cells transfected with mRNAs of wild, DS-Cav1, or LVRNA007 produced mixed populations of pre-fusion and post-fusion RSV F proteins that bound to the antigenic site IV antibody. In cells transfected with the wild-type mRNA, only 10% of cells expressed RSV F protein bound to site Φ antibody, suggesting that the wild-type mRNA mainly produced a post-fusion form of the F protein. In contrast, cells transfected with LVRNA007 mRNA were primarily bound to the antigenic site Φ and the trimeric site Φ antibody, suggesting that LVRNA007 mRNA was translated into trimeric pre-fusion F protein in vitro. Similarly to LVRNA007 mRNA, DS-Cav1 mRNA promoted pre-fusion and trimeric conformations. Western blotting demonstrated that the LVRNA007 construct successfully expressed F0 (F1 + F2), F0 dimer, and F0 trimer proteins in HEK293T cells (Figure 1C). The LVRNA007 mRNA was encapsulated into LNP, and the TEM image revealed that the spherical nanoparticles had a uniform size distribution (Figure 1D).

### 3.2. Durability of Cellular and Humoral Immune Responses After LVRNA007 Administration

**Figure 2:**
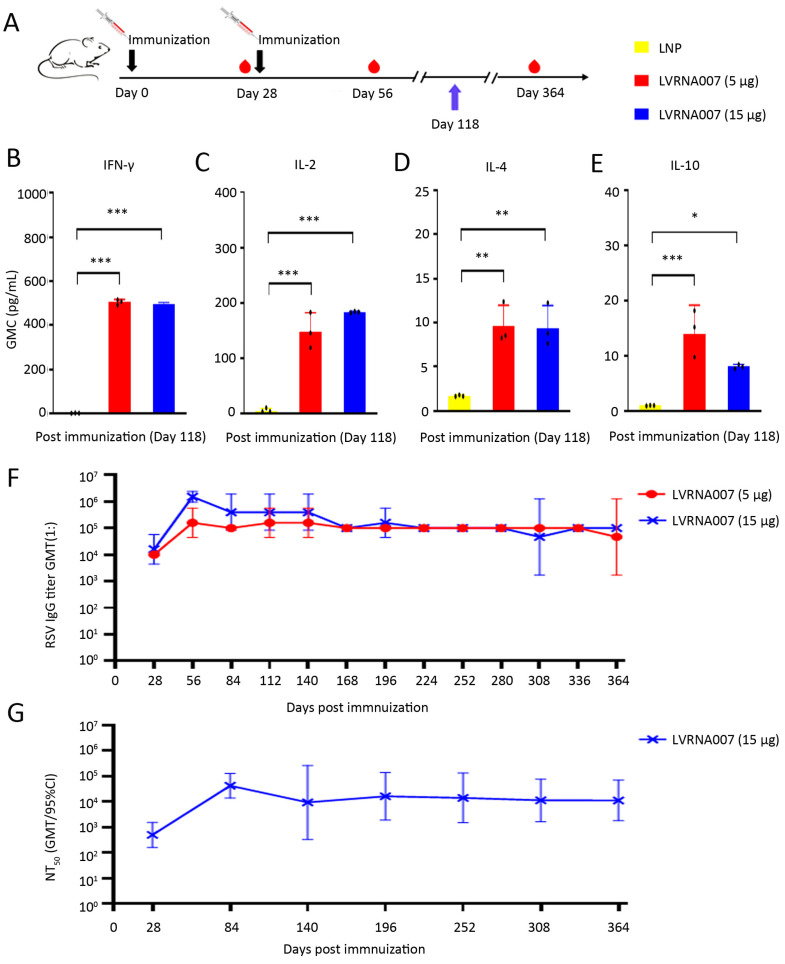
Durability of cellular and humoral immune responses after LVRNA007 administration. (**A**) Experimental design. Mice were immunized i.m. twice with LVRNA007 (5 μg or 15 μg, days 0 and 28) or LNP. (**B**–**E**) IFN-γ, IL-2, IL-4, and IL-10 cytokine levels in lymphocytes were detected through cytokine ELISAs on day 118 (90 days after the second vaccine dose). (**F**) IgG levels in serum samples were monitored for 364 days. (**G**) Neutralizing antibodies in serum samples were monitored for 364 days. *n* = 3. *, *p* < 0.05; **, *p* < 0.01; and ***, *p* < 0.001.

To evaluate the durability of LVRNA007-induced immune responses, mice were immunized i.m. with LVRNA007 (5 μg or 15 μg) or LNP (Figure 2A), followed by dynamic analysis of the cellular and humoral immune responses. ELISA revealed that LVRNA007 (5 μg and 15 μg) promoted IFN-γ, IL-2, IL-4, and IL-10 expression on day 118 (90 days after the second vaccine dose) (Figure 2B–E). Serum samples were collected every 28 days, and antibody tests were conducted. The results demonstrated that specific binding antibody titers maintained high levels at all time points (Figure 2F), as did the neutralizing antibody titers, which was as high as 1.29 × 10^4^ IU/mL at day 364 (Figure 2G). The results demonstrated that LVRNA007 could elicit strong cellular and humoral immune responses, which sustained high antibody levels for almost 1 year.

### 3.3. LVRNA007 Protected Mice from RSV Challenge Without Inducing VED

**Figure 3:**
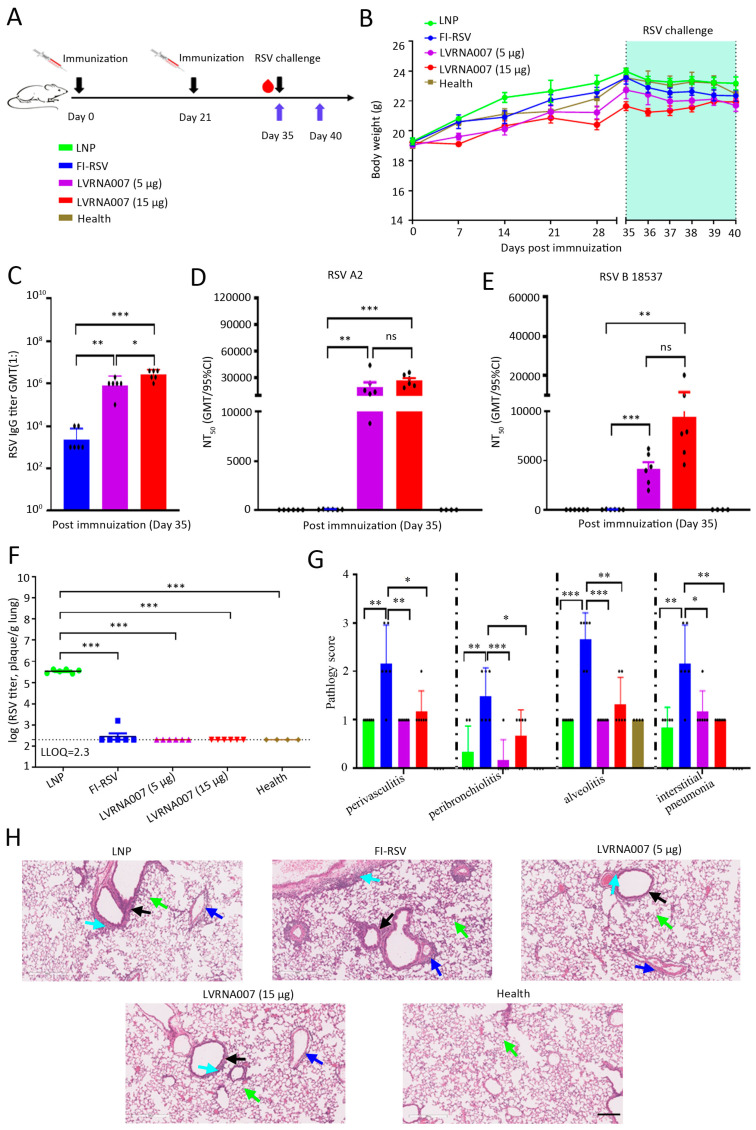
LVRNA007 protected mice from RSV challenge without inducing VED. (**A**) Schematic depiction of the experimental strategy. Mice were immunized i.m. on days 0 and 21 with LVRNA007 (5 μg or 15 μg), FI-RSV (0.05 μg), or LNP and challenged intranasally with 5 × 10^5^ PFU of RSV A2 on day 35. The Health group was not treated. (**B**) Body weight was recorded and diagramed for 40 days. (**C**) Specific binding antibody levels in the mice were analyzed using ELISA on day 35. (**D**,**E**) Neutralizing antibody titers against RSV A2 and RSV B 18537 strains were measured in serum samples on day 35. (**F**) RSV titer was measured in the lung tissue. (**G**) Pathological score was calculated on day 40. (**H**) Histopathological examination of lung tissue was conducted using HE staining. Scar bar, 200 μm. The blue arrows indicate perivasculitis. The black arrows indicate peribronchiolitis. The green arrows indicate alveolitis. The cyan arrows indicate interstitial pneumonia. *n* = 6. *, *p* < 0.05; **, *p* < 0.01; ***, *p* < 0.001; and ns, no significance.

To confirm the immunogenicity of LVRNA007, mice were immunized i.m. on days 0 and 21 with LVRNA007 (5 μg or 15 μg doses), FI-RSV, or LNP and challenged intranasally with RSV A2 on day 35 (Figure 3A). The body weight of the mice was monitored for the entire experimental period. We found that body weights in all groups increased before day 35. After the viral challenge, the body weights of all groups were reduced (Figure 3B). The binding antibody levels in mice that received a low dose (5 μg) or a high dose (15 μg) of LVRNA007 were higher than those in mice immunized with FI-RSV. High-dose LVRNA007 (15 μg) promoted higher antibody levels compared with low-dose LVRNA007 (5 μg) (Figure 3C). Neutralizing antibody titers against RSV A2 and RSV B 18537 strains were high in the low- (5 μg) and high-dose (15 μg) groups in comparison with the FI-RSV group on day 35. There was no significant change in neutralizing antibody titers in mice between the two LVRNA007-immunized groups (Figure 3D,E). The results indicated that LVRNA007 administered at a dose of 5 μg or 15 μg effectively induced high antibody production.

To evaluate the protection provided by LVRNA007, mice were challenged intranasally with RSV A2 on day 35. After the challenge with RSV A2, the lung tissues were collected from the vaccinated mice, and virus titers in the lungs were measured. The results demonstrated that all mice immunized with LVRNA007 (5 μg or 15 μg doses) or FI-RSV were almost clean of virus in the lung tissue (Figure 3F). A histopathological examination was used to evaluate each lung for evidence of perivasculitis, peribronchiolitis, alveolitis, or interstitial pneumonia. The results revealed that perivasculitis levels of mice in the FI-RSV-immunized group were significantly higher than those of mice in the LNP-immunized group. However, mice vaccinated with LVRNA007 (5 μg or 15 μg doses) had lower perivasculitis scores than mice immunized with FI-RSV (Figure 3G,H). Similar observations were made regarding peribronchiolitis, alveolitis, and interstitial pneumonia (Figure 3G,H). The findings suggested that LVRNA007 effectively prevented RSV infection in mice without inducing vaccine-enhanced disease (VED).

### 3.4. LVRNA007 Protected Cotton Rats from RSV Challenge Without Inducing VED

**Figure 4:**
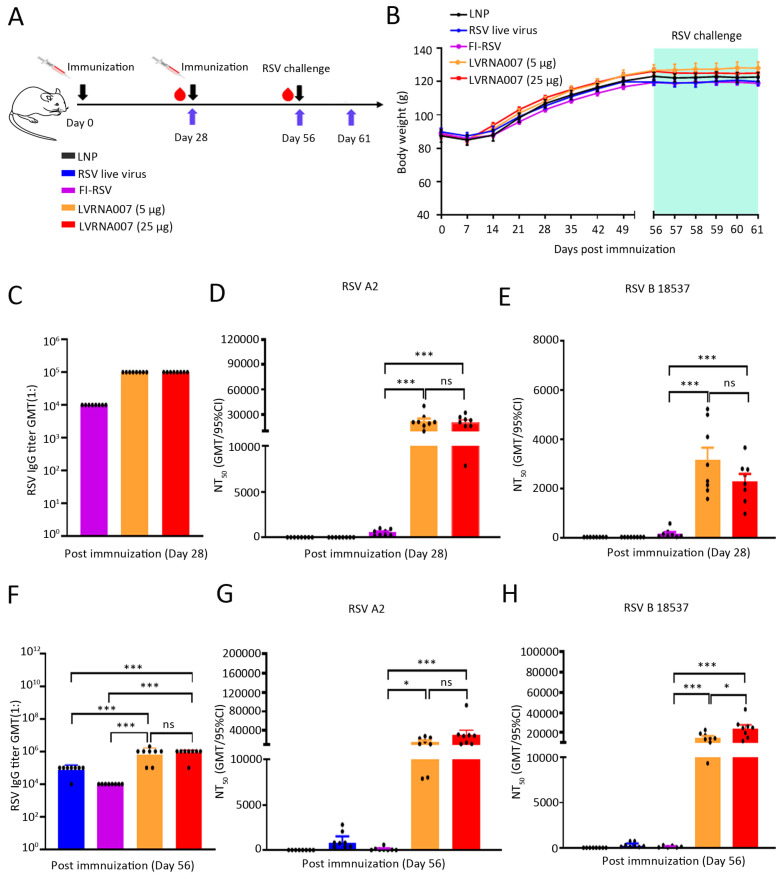
Effect of LVRNA007 on binding and neutralizing antibody titers in cotton rats. (**A**) Schematic depiction of the experimental strategy. Cotton rats were vaccinated i.m. on days 0 and 21 with LVRNA007 (5 µg or 25 µg), FI-RSV (0.05 μg), or LNP. The RSV live virus group received 5 × 10^5^ PFU of RSV A2 intranasally on day 28. All animals were challenged intranasally with 1 × 10^6^ PFU of RSV A2 on day 56. (**B**) Body weights of cotton rats were monitored and diagramed for 61 days. (**C**) Binding antibody levels in cotton rats were determined using ELISA on day 28. (**D**,**E**) Neutralizing antibody titers in serum against RSV A2 and RSV B 18537 strains were examined on day 28. (**F**) Binding antibody levels in cotton rats were measured using ELISA on day 56. (**G**,**H**) Neutralizing antibody titers in serum against RSV A2 and RSV B 18537 strains were analyzed on day 56. *n* = 8. *, *p* < 0.05; ***, *p* < 0.001; and ns, no significance.

Cotton rats have been reported to be semi-permissive to RSV infection. They can serve as an RSV VED model, and they can be used to assess the immunogenicity, efficacy, and safety of candidate RSV vaccines [54,55]. We therefore investigated the effects of LVRNA007 on cotton rats. Animals were administered LVRNA007 (5 µg or 25 µg, days 0 and 28), RSV live virus (day 28), FI-RSV (days 0 and 28), or LNP (days 0 and 28) and challenged with RSV on day 56 (Figure 4A). The body weight of all cotton rats increased before the challenge, but the trend stopped after the viral challenge (Figure 4B). Twenty-eight days post-immunization, the specific binding antibody levels were slightly higher in sera from LVRNA007 (5 µg or 25 µg)-immunized cotton rats than in sera from FI-RSV-immunized cotton rats (Figure 4C). However, there was a significant increase in neutralizing antibody titers against RSV A2 and RSV B 18537 strains in the LVRNA007 (5 µg or 25 µg)-immunized group compared with the FI-RSV-immunized group (Figure 4D,E). Furthermore, on day 56 (28 days after the second immunization), the specific binding antibody levels in the low- and high-dose groups were significantly higher than those in the FI-RSV-immunized or RSV live-virus-infected groups (Figure 4F). LVRNA007 (5 µg or 25 µg) significantly induced higher neutralizing antibody titers against RSV A2 and RSV B 18537 strains than FI-RSV on day 56 (Figure 4G,H). The results indicated that LVRNA007 effectively induced high levels of binding and neutralizing antibodies in cotton rats.

**Figure 5:**
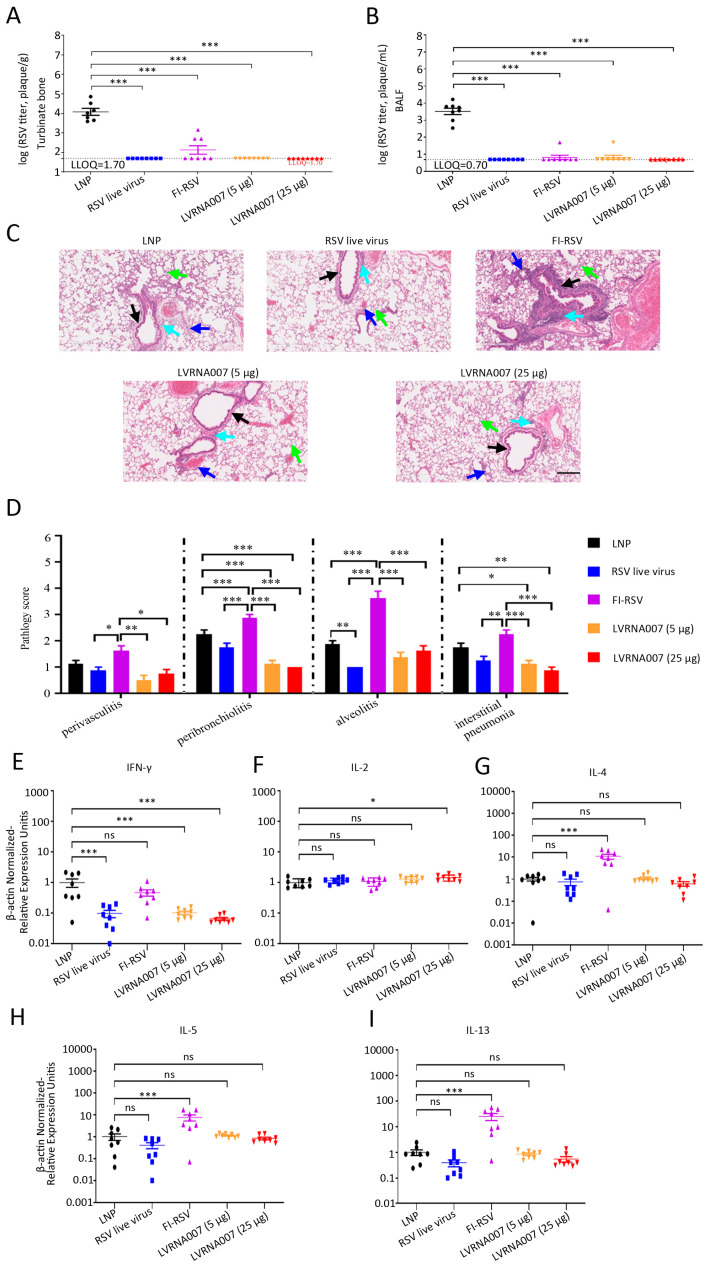
LVRNA007 protected cotton rats from RSV challenge without inducing VED. (**A**) RSV titer of turbinate bones in cotton rats following challenge on day 61. (**B**) RSV titer of lung washes in cotton rats on day 61. (**C**) HE staining was performed to identify histopathological changes in the lung tissue. Scar bar, 200 μm. The blue arrows indicate perivasculitis. The black arrows indicate peribronchiolitis. The green arrows indicate alveolitis. The cyan arrows indicate interstitial pneumonia. (**D**) Pathological score of the lung tissue was calculated on day 61. (**E**–**I**) IFN-γ, IL-2, IL-4, IL-5, and IL-13 cytokine mRNA levels in the lung tissue were examined using real-time PCR. *n* = 8. *, *p * < 0.05; **, *p* < 0.01; ***, *p * < 0.001; and ns, no significance.

To further evaluate the effects of LVRNA007 on VED, cotton rats were challenged with RSV on day 56. Five days post-challenge, turbinate bone and lung tissue samples were collected and used for RSV titers. The results revealed that RSV titers of the turbinate bone and lung washes from the LNP-immunized group were significantly higher than those of the other groups (Figure 5A,B), suggesting that LVRNA007 (5 µg or 25 µg), RSV live virus, and FI-RSV immunization remarkably decreased RSV titers in the noses and lung tissue. Perivasculitis, peribronchiolitis, alveolitis, and interstitial pneumonia were analyzed using HE staining. Compared with LVRNA007-immunized animals, FI-RSV-immunized animals exhibited significant perivasculitis, peribronchiolitis, alveolitis, and interstitial pneumonia following the RSV challenge. Moreover, cotton rats vaccinated with LVRNA007 (5 µg or 25 µg) had lower pathology scores of peribronchiolitis and interstitial pneumonia than those immunized with LNP (Figure 5C,D). Furthermore, cytokine levels in the lung tissue revealed that the IFN-γ mRNA level was lower in LVRNA007 (5 µg or 25 µg)-immunized cotton rats than in LNP- or FI-RSV-immunized cotton rats on day 5 post-challenge (Figure 5E). No significant difference between any of the groups was observed in IL-2 mRNA levels (Figure 5F). The expression of IL-4, IL-5, and IL-13 mRNA was significantly higher in the FI-RSV-immunized group than in the LNP-immunized group (Figure 5G–I). However, no differences in IL-4, IL-5, or IL-13 mRNA levels were observed between the LVRNA007-immunized groups and the LNP-immunized group (Figure 5G–I). The findings implied that LVRNA007 effectively protected against RSV challenge, prevented inflammation, and did not cause VED in cotton rats.

## 4. Discussion

In this study, we developed LVRNA007 encoding the RSV F protein stabilized in a pre-fusion conformation. The durability of the cellular and humoral immune response following two doses of LVRNA007 was assessed in mice. In addition, immunogenicity and protection were evaluated in mice and cotton rats. We found that LVRNA007 effectively induced high levels of binding and neutralizing antibodies, provided protection against RSV challenge, and it did not cause VED in mice or cotton rats.

The RSV F protein is cleaved by Furin-like proteases into F1, peptide 27, and F2, which form a trimer of F1-F2 heterodimers. Atomic-level structures of the RSV F protein have exhibited two distinct conformations, pre-fusion and post-fusion [53,56,57]. Stabilized pre-fusion immunogens have been found to promote neutralizing antibody responses in mice and macaques, with titers at the high end of those achieved after recurrent natural infections in humans [45]. RSV-neutralizing antibodies targeting antigenic site Φ, which is only present in the pre-fusion conformation of the protein, are 10–100-fold more potent than palivizumab [45]. Previous studies reported that the mRNA vaccines have mainly concentrated on pre-fusion conformations of the RSV F protein [58,59]. In this study, we optimized DS-Cav1, described by McLellan et al. [45], to design LVRNA007, which stabilized RSV F in its pre-fusion conformation and retained antigenic site Φ. The Furin cleavage sites were removed in the LVRNA007 construct; therefore, our in vitro study showed that F0, not F1, protein expression was induced by the LVRNA007 construct in HEK293T cells. Moreover, the F0 dimer and F0 trimer were produced in the LVRNA007 construct in HEK293T cells. Multiple studies have proven that multimeric antigen could elicit superior immunogenicity compared to monomeric antigen [60,61,62,63], suggesting that the multimerization of LVRNA007 can promote immunogenicity in the current study. The durability of the immune response after vaccination is important. The durability study of the SARS-CoV-2 mRNA vaccination showed that immune responses lasted for 6–9 months [64]. In the current study, we found that LVRNA007-induced cellular immune responses lasted for at least 118 days, and neutralizing antibody levels maintained high levels for almost 1 year, thus presenting excellent durability of the cellular and humoral immune responses after LVRNA007 administration. In addition, it is preferred that a meaningful RSV vaccine provides cross-strain protection. In this study, we demonstrated that although the protein sequence is based on RSV A strain, LVRNA007 immunization elicited high levels of neutralizing antibody titers against both RSV A2 and RSV B 18537 strains. We also found that RSV live and FI-RSV groups showed minimal levels of neutralizing antibody, but with titers very low in comparison with those induced by LVRNA007. A detailed mechanism of protection in RSV live and FI-RSV groups remains unclear, but it is possible that cellular immunity and non-neutralizing antibodies play a role in this animal model. 

Safety is always a significant challenge in the development of an RSV vaccine. The failure of FI-RSV vaccines exemplified this concern, as FI-RSV vaccination in naïve infants induced VED, which led to significant increases in hospitalization and death following RSV infection [65]. In the current study, although the virus load was undetectable in both LVRNA007- and FI-RSV-immunized groups, animals vaccinated with LVRNA007 had significantly lower lung pathology scores than those immunized with FI-RSV following the RSV challenge. The lung pathology was low in LVRNA007-immunized animals after the RSV challenge, suggesting that LVRNA007 avoided the risk of VED. LNPs, as a vital component of the COVID-19 mRNA vaccines, play a key role in protecting and transporting mRNA to cells [66]. Recently, the ionizable cationic lipid ALC-0315 in a COVID-19 mRNA vaccine was found to have apparent cytotoxicity and intrinsic cytotoxicity [67]. Therefore, the effects of LNPs should be considered. In our studies, pathological scores in the LNP group were low, suggesting that inflammation was mild.

CD4+ T-helper (Th) cells in both mice and humans represent a functionally heterogeneous population including at least two distinct subsets, Th1 and Th2 cells. Th1 cells promote macrophage activation by secreting IL-2, IFN-γ, and tumor necrosis factor-beta (TNF-β). Th2 cells, which produce IL-4, IL-5, IL-6, IL-10, and IL-13, promote antibody production and contribute to the humoral response [68,69,70,71]. Polarization of the CD4+ T-cell response to a Th2 phenotype was associated with VED following FI-RSV immunization in mouse models [65,72]. In our studies with LVRNA007, cytokine examination following RSV challenge revealed that both the low and high doses of LVRNA007 caused no significant change in the expression of Th2-associated cytokines, including IL-4, IL-5, and IL-13, whereas FI-RSV significantly promoted the expression of these cytokines. Additionally, RSV infection upregulated IFN-γ expression in both unvaccinated cotton rats and those vaccinated with FI-RSV. The findings indicated that administration of LVRNA007 prevented RSV infection and an RSV-infection-related inflammatory response. Currently, studies of RSV mRNA vaccines are primarily aimed at adults and the elderly [39,59,73]. Evaluation is still lacking for the immunogenicity, efficacy, and safety of RSV mRNA vaccines in infants, indicating that the immunogenicity, efficacy, and safety of LVRNA007 in neonatal animals will be necessary to assess in the future. Moreover, clinical studies are needed to evaluate the efficacy and safety of LVRNA007 in humans.

## 5. Conclusions

We successfully developed an mRNA vaccine, LVRNA007, encoding the RSV F protein stabilized in its pre-fusion conformation. The LVRNA007-induced immune responses lasted as long as almost 1 year in mice. Immunization with two doses of LVRNA007 achieved high levels of both binding and neutralizing antibodies in mice and cotton rats. The efficacy and safety studies revealed that LVRNA007 protected rodents against RSV challenge without causing VED. Therefore, LVRNA007 is a potential vaccine candidate for RSV infection control.

## Data Availability

The original contributions presented in this study are included in the article. Further inquiries can be directed to the corresponding author.
